# Prevalence and factors associated with severe depressive symptoms in older west African people living with HIV

**DOI:** 10.1186/s12888-020-02837-0

**Published:** 2020-09-10

**Authors:** Charlotte Bernard, Hélène Font, Zélica Diallo, Richard Ahonon, Judicaël Malick Tine, Franklin N’guessan Abouo, Aristophane Tanon, Eugène Messou, Moussa Seydi, François Dabis, Nathalie de Rekeneire, the IeDEA West Africa Cohort Collaboration, Marcel Djimon Zannou, Armel Poda, Fred Stephen Sarfo, Eugene Messou, Henri Chenal, Kla Albert Minga, Emmanuel Bissagnene, Aristophane Tanon, Moussa Seydi, Akessiwe Akouda Patassi, Sikiratou Adouni Koumakpai-Adeothy, Lorna Awo Renner, Sylvie Marie N’Gbeche, Clarisse Amani Bosse, Kouadio Kouakou, Madeleine Amorissani Folquet, François Tanoh Eboua, Fatoumata Dicko Traore, Elom Takassi, François Dabis, Elise Arrive, Eric Balestre, Renaud Becquet, Charlotte Bernard, Shino Chassagne Arikawa, Alexandra Doring, Antoine Jaquet, Karen Malateste, Elodie Rabourdin, Thierry Tiendrebeogo, Sophie Desmonde, Julie Jesson, Valeriane Leroy, Didier Koumavi Ekouevi, Jean-Claude Azani, Patrick Coffie, Abdoulaye Cissé, Guy Gnepa, Apollinaire Horo, Christian Kouadio, Boris Tchounga

**Affiliations:** 1 Bordeaux Population Health Research Center, Univ. Bordeaux, Inserm, UMR 1219, F-33000 Bordeaux, France; 2 Bordeaux Population Health Research Center, Univ. Bordeaux, ISPED, UMR 1219, F-33000 Bordeaux, France; 3 Service de maladies infectieuses et tropicales, CHU Treichville, Abidjan, Côte d’Ivoire; 4 Centre de prise en charge de recherche et de formation (CePReF), Yopougon Attié Hospital, Abidjan, Côte d’Ivoire; 5 Service de maladies infectieuses et tropicales, CRCF, CHNU de Fann, Dakar, Senegal

**Keywords:** HIV, Aging, Depression, Sub-Saharan Africa

## Abstract

**Background:**

Depression is one of the most common psychiatric disorders in people living with HIV (PLHIV). Depression has a negative impact on both mental and physical health and is mainly associated with suboptimal HIV treatment outcomes. To encourage successful aging and the achievement of the 3 × 90 objectives in older PLHIV, the psychological domain must not be neglected. In this context and as data are scarce in West Africa, this study aimed to evaluate the prevalence and the factors associated with severe depressive symptoms in older PLHIV living in this region of the world.

**Methods:**

Data from PLHIV aged ≥50 years and on ART since ≥6 months were collected in three clinics (two in Côte d’Ivoire, one in Senegal) participating in the West Africa International epidemiological Databases to Evaluate AIDS (IeDEA) collaboration. The severity of depressive symptoms was measured using the Center for Epidemiological Studies Depression scale (CES-D), and associated factors were identified using logistic regressions.

**Results:**

The median age of the 334 PLHIV included in the study was 56.7 (53.5–61.1), 57.8% were female, and 87.1% had an undetectable viral load. The prevalence of severe depressive symptoms was 17.9% [95% Confidence Interval (95% CI): 13.8–22.0]. PLHIV with severe depressive symptoms were more likely to be unemployed (adjusted Odd Ratio (aOR) = 2.8; 95% CI: 1.4–5.7), and to be current or former tobacco smokers (aOR = 2.6; 95% CI: 1.3–5.4) but were less likely to be overweight or obese (aOR = 0.4; 95% CI: 0.2–0.8).

**Conclusions:**

The prevalence of severe depressive symptoms is high among older PLHIV living in West Africa. Unemployed PLHIV and tobacco smokers should be seen as vulnerable and in need of additional support. Further studies are needed to describe in more details the reality of the aging experience for PLHIV living in SSA. The integration of screening and management of depression in the standard of care of PLHIV is crucial.

## Background

During the two last decades, a dramatic positive change has been observed worldwide in the demographics of people living with HIV (PLHIV) [1]. Increased access and use of HIV medical services and the improvement in antiretroviral therapy (ART) access are the two main reasons for this change. HIV infection is now considered a chronic disease in most settings, including in sub-Saharan Africa (SSA) [1]. PLHIV have a lifespan comparable to that observed in the general population and are getting older.

With aging, PLHIV experience more complications in the physiological domain. The compromised immune system with the involution of the thymus, ART side effects, and polypharmacy are a pathway to explain these complications [2]. Older patients receiving ART are at an increased risk of age-associated non-communicable comorbidities (AANCC), like cardiovascular diseases, renal diseases, diabetes, cancers, osteoporosis, and neuropsychological impairment [3, 4]. Among these AANCC, insufficient attention has been paid to mental health issues. However, to facilitate successful aging in the context of chronic diseases, such as HIV disease, the psychological and social domains must not be neglected [5]. In Young et al. model [5], psychological and social domains could be a way to compensate for physiological limitations (i.e., multiple chronic conditions) and allow, even in the context of disease and disability, to experience a good quality of life.

In PLHIV on ART, depression is among the most common psychiatric disorders [6] and has a high prevalence. In SSA, a recent meta-analysis reported a pooled prevalence of 13% for major depressive disorder and between 14 to 32% for severe depressive symptoms in those patients [7]. The consequences of depression on linkage to care and HIV response are crucial. First, depression has been shown to predict non-adherence to ART [8–11]. In South Africa, one study reported that, compared to adherent patients, non-adherent patients had a 3-fold higher risk of presenting moderate to severe depressive symptoms [12]. Second, depression is associated with poor health status overall [13–16], and poor HIV outcomes [17]: low CD4 progression [18–20], but also with faster progression to AIDS and increased mortality [6, 21]. As in the general population, depression is highly associated with suicide [22]. Third, depression impacts the quality of life in PLHIV [6]. Its occurrence leads to alteration of economic productivity, unemployment [20, 23], social isolation, stigmatization [24], disability [25], and also high sexual risk behaviors [26]. In western countries, untreated depression in PLHIV has also been related to increased cognitive complaints and negative consequences in multiple aspects of quality of life [27–29]. In addition to aging-related problems, these medical and psychological factors may be exacerbated in older PLHIV [30]. In this context, depression could seriously compromise ART outcomes at individual and population levels and the achievement of the 3 × 90 objectives.

Despite depression substantially increasing over the past few years, data to document this significant dimension of the HIV epidemic in older PLHIV in SSA are scarce. While SSA is the part of the world that is the most affected by HIV/AIDS [6], depression often remains under-diagnosed [6, 31]. As depression in PLHIV emerges as a public health issue, the risk of a burden on the healthcare system and human resources is significant. Evaluating the prevalence of severe depressive symptoms and identifying associated factors will provide insights for clinical interventions. In this context, the present study aims to describe the prevalence and identify factors associated with severe depressive symptoms in PLHIV aged 50 years and above living in West Africa.

## Methods

### Study design

This study is a part of an ancillary study project within the West Africa network of the International epidemiological Databases to Evaluate AIDS (IeDEA) of the US National Institutes of Health (https://www.iedea.org/regions/west-africa/) [32]. This study was conducted in two different countries, in three urban clinics with a large caseload of PLHIV and selected by convenience: the infectious and tropical disease department of the Treichville University Hospital (Abidjan, Côte d'Ivoire), the public referral clinic (CePReF) in Yopougon Attié Hospital (Abidjan, Côte d’Ivoire) and the infectious and tropical disease department of the Fann University Hospital (Dakar, Senegal). The inclusion period of the study occurred between February 2016 to November 2017. We performed a cross-sectional analysis based on the baseline data of a 2-year longitudinal study evaluating different aspects of aging with HIV (cognition, physical function, and frailty), and the presence of severe depressive symptoms with a follow-up still ongoing.

### Study population

Patients were recruited at the time of their usual HIV follow-up visit. Patients were eligible if they were living with type-1 HIV, 50 years old or older, and on ART for at least six months. We excluded patients having a psychiatric illness (including being under psychotropic treatment), a history of opportunistic cerebral infection, a neurological pathology (history of stroke or Parkinson disease), a current disabling opportunistic infection, meningitis, a sensitivo-motor paralysis, or cancer under treatment or respiratory or cardiac insufficiency.

### Severity of depressive symptoms

The severity of depressive symptoms was evaluated with the Center for Epidemiological Studies Depression scale (CES-D), a 20-item self-report scale assessing the occurrence of depressive symptoms during the past week using a 4-point Likert scale [33]. Due to variability in patients’ literacy level, each item was read aloud by a trained doctor or nurse. A translation of items in the national language other than French was used if necessary. Although CES-D does not provide a clinical diagnosis of depression, this scale has been reported as suitable in the context of epidemiological studies targeting French-speaking patients [34]. Even though this scale was not validated specifically in older PLHIV in West Africa, a recent meta-analysis reported a high pooled sensitivity and specificity for the CES-D, based on three African studies (Uganda, Zambia, South Africa) for threshold ranges between 16 and 22 (sensitivity 82% [95% CI, 73–87], specificity 73% [95% CI, 63–80]) [35]. It was also validated in Uganda, with scores > 16 having a good sensibility and specificity in predicting major depression disorders [36]. For the analyses, a total score ≥ 17 for men and ≥ 23 for women was used to define severe depressive symptoms [37].

### Other covariates

Data were collected through essential questions and medical examination. Patients’ sociodemographic characteristics as age, gender, level of education, marital status, and employment status were recorded.

Concerning HIV medical data, the initial clinical stage was defined using the Centers for Disease Control and Prevention (CDC) definition (A, B, or C). Baseline Nadir CD4 and more recent CD4 were presented in two categories (≤200 vs > 200 cells/μl, and < 500 vs ≥500 cells/μl, respectively). The composition of the initial and current ART treatment was presented through a categorical variable (TDF/3TC/EFV vs other combination). The duration of HIV disease was calculated as the delay in months between the first positive serology date and the study’s inclusion date. Adherence to ART was defined as the percentage of tablets the patient declared to take over 7 days (in comparison to the prescribed total number of tablets over this period).

Substance use was evaluated through basic questions for tobacco and drugs (current, former, or never). Alcohol consumption was evaluated with the AUDIT-C. A score ≥ 3 for women or ≥ 4 for men was considered as hazardous drinking.

The Body Mass Index (BMI) was calculated as weight in kilograms/height in meters squared and considered in two categories: low or normal BMI (low when < 18 kg/m^2^, normal between 18 to 24 kg/m^2^) and high when ≥25 kg/m^2^. Patients were also asked if they had ever been diagnosed with these comorbidities: hypertension, diabetes, hyperlipidemia, C or B hepatitis, tuberculosis, migraine, arthrosis, or other. A variable “comorbidities” was created with three categories: absence, only one, or more than one comorbidity. History of trauma and neurologic diseases was also documented.

### Measures of functional status

Activities of the daily living (ADL) [38] and Instrumental activities with daily living (IADL) [39] scales were used to evaluate the autonomy of the patients. The final ADL and IADL scores range from 0 to 6 and 0 to 4, respectively (0, indicating the lowest degree of autonomy).

### Statistical analysis

The study sample characteristics were described using median and interquartile range (IQR) for continuous variables, numbers, and proportions for categorical variables.

In order to describe the five most reported depressive symptoms among depressed PLHIV, we used the factorial structure described by Sheehan et al., [40] grouping the items 1, 2, 5, 7, 11, 13 and 20 as somatic symptoms, the items 3, 6, 14, 17 et 18 as depressive affects, the items 4, 8, 12 and 16 as negative affects, and the items 9, 10, 15 et 19 as interpersonal deficit. For this analysis, each item was recoded in two categories: the significant presence of the symptoms (≥3 days) or no (≤2 days). As the expression of depression could be different between females and males, we identified which symptoms were significantly most reported according to gender, using Chi-2 tests.

The prevalence of severe depressive symptoms was reported. Then, factors associated with the presence of severe depressive symptoms were evaluated using logistic regression analyses. Before conducting logistic regression analyses, a multivariable Random Forest imputation of missing data was performed. As no significant difference was observed between the two databases (with and without missing data), we used the database with imputed missing data for these analyses. In the multivariable regression model, we included all variables associated with the dependent variable with a *p*-value≤0.2 in univariable analyses. The “inclusion centers” variable was included as a cofounder in each model. Unbalanced variables (85%/15%) were excluded from the multivariable analyses. The final model was obtained with a backward selection, and we considered significant associations at *p* < 0.05. The goodness of fit of the final model was evaluated with the Hosmer-Lemeshow test (*p* > 0.05). Statistical analyses were computed using R software.

### Ethical consideration

Ethical clearance was obtained from the national ethics committee of each participating country (Senegal: Conseil National d’Ethique de la Recherche en Santé (CNERS); Côte d’Ivoire: Comité National de l’Ethique et de la Recherche). All the patients gave their written consent before being included in the study. Participants’ right to refuse the participation was kept, and the confidentiality of the patient was maintained. To have a further assessment on their condition, patients with severe depressive symptoms were referred to a psychiatrist within the hospital.

## Results

### Characteristics of the sample

A total of 334 patients were included in our study. The median (IQR) age was 56.7 (53.5–61.1) years. Among them, 34.7% were aged 60 and older (only 11 were aged ≥70), 57.8% were female, and 50.6% had a primary level of education or less. Almost half of them lived in couple (46.4%) and were currently employed (53.6%) (Table 1).

**Table 1:** Characteristics of the study population according to the severity of depressive symptoms

Characteristics	No severe depressive symptoms		Severe depressive symptoms		p*	TOTAL	
	N	%	N	%		N	%
	274	(82.1)	60	(17.9)		334	(100.0)
**Socio-demographic data**							
Age					0.73		
50–59	180	(65.7)	38	(63.3)		218	(65.3)
60–69	94	(34.3)	22	(36.7)		116	(34.7)
Gender					0.05		
Male	109	(39.8)	32	(53.3)		141	(42.2)
Female	165	(60.2)	28	(46.7)		193	(57.8)
Education level					0.13		
No education	81	(29.6)	10	(16.7)		91	(27.2)
Primary school	61	(22.3)	17	(28.3)		78	(23.4)
Secondary school	100	(36.5)	28	(46.7)		128	(38.3)
University & Tertiary Education	32	(11.6)	5	(8.3)		37	(11.1)
Marital Status					0.36		
Married/Cohabitating	125	(45.6)	30	(50.0)		155	(46.4)
Widowed	93	(34.0)	14	(23.3)		107	(32.0)
Divorced	20	(7.3)	7	(11.7)		27	(8.1)
Never married	36	(13.1)	9	(15.0)		45	(13.5)
Employment status					0.08		
Currently employed	153	(55.8)	26	(43.3)		179	(53.6)
Unemployed	70	(25.5)	22	(36.7)		92	(27.5)
Retired	51	(18.6)	12	(20.0)		63	(18.9)
**HIV Clinical data**							
Clinical disease stage at ART initiation					0.42		
A	86	(31.4)	14	(23.3)		100	(29.9)
B	145	(52.9)	37	(61.7)		182	(54.5)
C - AIDS	39	(14.2)	9	(15.0)		48	(14.4)
Unknowm	4	(1.5)	.	.		4	(1.2)
Duration of infection (months)					0.08		
[6–69[	75	(27.4)	8	(13.3)		83	(24.9)
[69–108[	69	(25.2)	14	(23.3)		83	(24.9)
[108–141[	65	(23.7)	16	(26.7)		81	(24.3)
≥ 141	65	(23.7)	22	(36.7)		87	(26.0)
Nadir CD4					**0.02**		
> 200	106	(38.7)	14	(23.3)		120	(35.9)
≤ 200	158	(57.7)	45	(75.0)		203	(60.8)
Missing	10	(3.6)	1	(1.7)		11	(3.3)
Most recent CD4					0.38		
≥ 500	141	(51.5)	27	(45.0)		168	(50.3)
< 500	130	(47.4)	32	(53.3)		162	(48.5)
Missing	3	(1.1)	1	(1.7)		4	(1.2)
Detectable Viral load	27	(9.9)	16	(26.7)	**< 0.001**	43	(12.9)
Missing	47	(17.2)	9	(15.0)		56	(16.8)
Initial ART combinaison					0.11		
3TC + TDF + EFV	72	(26.3)	10	(16.7)		82	(24.6)
Other	200	(73.0)	50	(83.3)		250	(74.9)
Missing	2	(0.7)	.	.		2	(0.6)
Actual ART combinaison					0.06		
3TC + TDF + EFV	151	(55.1)	25	(41.7)		176	(52.7)
Other	123	(44.9)	35	(58.3)		158	(47.3)
Adherence to ART (yes)	261	(95.3)	54	(90.0)	0.06†	315	(94.3)
**Substance use**							
Hazardous drinkers	18	(6.6)	7	(11.7)	0.18	25	(7.5)
Tobacco use (current/former)	39	(14.2)	18	(30.0)	**0.004**	57	(17.1)
Drug consumption	4	(1.5)	2	(3.3)	0.30†	6	(1.8)
**Anthropometric and medical data**							
Overweight/obesity	114	(41.6)	13	(21.7)	**0.004**	127	(38.0)
Comorbidities					0.61		
None	119	(43.4)	23	(38.3)		142	(42.5)
One	97	(35.4)	21	(35.0)		118	(35.3)
More than one	58	(21.2)	16	(26.7)		74	(22.2)
History of trauma (yes)	18	(6.6)	4	(6.7)	1.0	22	(6.6)
History of neurological disease (yes)	37	(13.5)	11	(18.3)	0.33	48	(14.4)
**Inclusion centers**					0.31		
CePREF, Abidjan	93	(33.9)	15	(25.0)		108	(32.3)
ITDD, Abidjan	140	(51.1)	37	(61.7)		177	(53.0)
ITDD, Dakar	41	(15.0)	8	(13.3)		49	(14.7)

Abbreviations: *ART* antiretroviral therapy, *ITDD* infectious and tropical disease department

* *p*-value associated with Chi-2 test

† *p*-value associated with Exact Fisher Test

A large majority of patients had an undetectable viral load (87.1%); half of them having CD4 ≥ 500 (50.3%) and 60.8% a Nadir CD4 < 200. The median (Interquartile - IQR) duration of HIV infection was 108 months (68.9–141.0). Fourteen percent (14.4%) were on stage C at ART initiation. Concerning ART treatment, 25 and 52.7% received the standard combination for their initial and current treatment (according to the national treatment guidelines), respectively. Patients reported high adherence to ART (94.3%).

Few patients reported substance use (< 8%), except for tobacco (current/previous) (17.1%).

For other medical issues, 38% were overweight or obese, 35.3% reported one comorbidity in addition to their HIV disease, and 22.2% more than one.

In terms of the ADL and IADL instruments, 97.0 and 99.1% of the patients got the maximum score (6 or 4, respectively).

### Prevalence of severe depressive symptoms

**Fig. 1:**
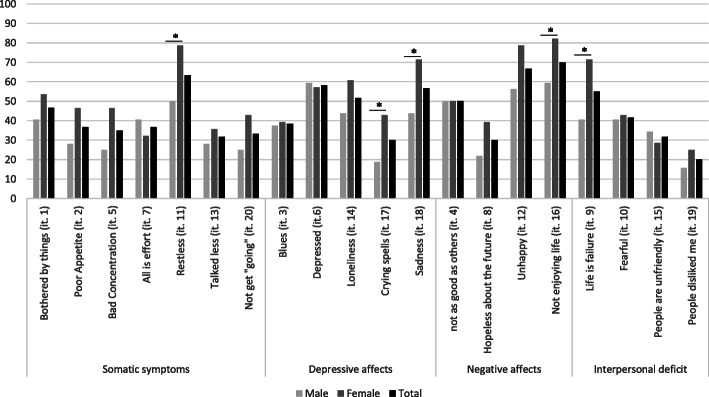
Frequency of depressive symptoms reported by the patients in the whole sample and according to gender repartition. *Significant difference between male and female

The prevalence of severe depressive symptoms was 17.9% [95% Confidence Interval (CI): 13.8–22.0]. Among PLHIV with severe depressive symptoms (*N* = 60), 80% reported somatic symptoms, 73.3% depressive affects, 71.7% negative affects and 40% interpersonal deficit. The five most reported symptoms (Fig. 1) were: “not enjoying life” (70%), “being unhappy” (66.7%), “being restless” (63.3%), “feeling depressed” (58.3%) and “sadness” (56.7%). Compared to males, females reported more frequently the following symptoms: “being restless” (78.6% vs 50%, *p* = 0.02), “crying spells” (42.9% vs 18.7%, *p* = 0.03), “sadness” (71.4% vs 43.7%, *p* = 0.03) and “not enjoying life” (82.1% vs 58.4%, *p* = 0.05). They also reported more frequently that their life is a failure (71.4% vs 40.6%, *p* = 0.02).

### Factors associated with severe depressive symptoms

In univariate models (Table 2), having a longer duration of the disease ≥141 months (OR = 3.2; 95% CI: 1.3–7.6), being unemployed (OR = 1.9; CI95%: 1.0–3.8), a Nadir CD4 ≤ 200 (OR = 2.1; CI95%: 1.1–4.2), a detectable viral load (OR = 3.1; 95% CI: 1.5–6.3) were significantly associated with the presence of severe depressive symptoms. PLHIV with severe depressive symptoms were also more likely to be current or former tobacco smokers (OR = 2.5; 95% CI: 1.3–4.9) but were less likely to be overweight or obese (OR = 0.4; 95% CI: 0.2–0.8).

**Table 2:** Factors associated with severe depressive symptoms in the study population

Variables		Univariable models		Multivariable model	
	% severe depressive symptoms^a^	OR (95% CI)*	*p*-value	aOR (95% CI)*	*p*-value
Age			0.71		
50–59 years old	17.4	1			
≥ 60 years old	18.9	1.1 (0.6–2.0)			
Gender			0.09		
Men	22.7	1			
Women	14.5	0.6 (0.4–1.1)			
Education level					
No education	10.9	1			
Primary school	21.8	2.2 (0.9–5.2)	0.08		
Secondary school	21.9	2.1 (0.9–4.7)	0.07		
University & Tertiary Education	13.5	1.1 (0.3–3.6)	0.87		
Marital Status					
Married/Cohabitating	19.3	1			
Widowed	13.1	0.7 (0.3–1.3)	0.23		
Divorced	25.9	1.6 (0.6–4.2)	0.33		
Never married	20.0	1.0 (0.4–2.3)	0.99		
Employment status			0.11		**0.01**
Currently employed	14.5	1		1	
Unemployed	23.9	1.9 (1.0–3.8)	**0.04**	2.8 (1.4–5.7)	**0.003**
Retired	19.1	1.3 (0.6–2.8)	0.51	1.2 (0.5–2.6)	0.67
Duration of HIV infection					
[6–69[	9.6	1			
[69–108[	16.7	1.9 (0.8–4.8)	0.17		
[108–141[	19.7	2.3 (0.9–5.8)	0.07		
≥ 141	24.4	3.2 (1.3–7.6)	**0.01**		
Clinical disease stage					
A	14.0	1			
B	19.9	1.3 (0.6–2.7)	0.46		
C	18.7	1.2 (0.5–3.2)	0.67		
Nadir CD4			**< 0.0001**		
> 200	11.2	1			
≤ 200	22.0	2.1 (1.1–4.2)			
CD4			0.36		
≥ 500	15.9	1			
< 500	20.1	1.3 (0.7–2.3)			
Viral load			**0.002**		
Undetectable	15.1	1			
Detectable	37.2	3.1 (1.5–6.3)			
Initial ART combination			0.10		
3TC + TDF + EFV	12.2	1			
Other	19.8	1.8 (0.9–3.8)			
Actual ART combination			0.06		
3TC + TDF + EFV	14.2	1			
Other	22.1	1.7 (0.9–3.1)			
Non adherence to ART (ref.: adherent)	38.5	3.1 (0.9–9.9)	0.06		
Alcohol consumption (ref.: no)^b^	28.0	1.9 (0.7–4.8)	0.18		
Tobacco (current/former) (ref.: no)	31.6	2.5 (1.3–4.9)	**0.01**	2.6 (1.3–5.4)	**0.01**
Drug consumption (ref.: no)	33.3	1.9 (0.3–11.0)	0.45		
BMI			**0.01**		**0.01**
Normal / underweight	22.7	1		1	
Overweight/obesity	5.9	0.4 (0.2–0.8)		0.4 (0.2–0.8)	
Comorbidities					
No	16.2	1			
Only one	17.8	1.2 (0.6–2.3)	0.62		
More than one	21.6	1.7 (0.8–3.4)	0.18		
History of trauma (ref.: no)	18.2	0.9 (0.3–3.1)	0.99		
History of neurological disease (ref.: no)	22.9	2.3 (0.9–5.6)	0.07		

Abbreviations: *aOR* adjusted Odd Ratio, *ART* antiretroviral therapy, *BMI* Body Mass Index, *CI* Confident Interval, *EFV* Efavirenz, *OR* odd ratio, *ref.* Reference group, *TDF* Tenofovir, *3TC* Lamivudine

*results considered as significant (*p* < 0.05) (bold text)

^a^Calculated after imputation of missing data

^b^ref.: no: means that the OR is computed taking this category “absence of this medical problem” as the reference group

In the multivariate model, being unemployed (adjusted OR (aOR) = 2.8; 95% CI: 1.4–5.7), being a current or former tobacco smokers (aOR = 2.6; 95% CI: 1.3–5.4) and being overweight or obese (aOR = 0.4; 95% CI: 0.2–0.8) remained associated with severe depressive symptoms (Goodness of Fit: χ^2^ = 5.4, *p* = 0.71).

## Discussion

In the present study, in a large sample of PLHIV aged 50 years and above, severe depressive symptoms were observed in almost 18% of the patients. The severity of depressive symptoms seems to be more related to social (i.e., having no professional activity) and behavioral (i.e., being current or former tobacco) aspects. Unexpectedly, being overweight or obese seems to be a protective factor for the occurrence of depressive symptoms.

The prevalence of depressive symptoms is high and could not be neglected. In the context of the 3 × 90 objectives, screening, and management of mental health disorders, including depression, has been listed as a research priority to improve timely diagnosis, ART initiation, retention, and viral suppression [41]. Recent data have also reported a 24% increased risk of mortality in older PLHIV who are depressed [42].

Recent publications from western countries reported variable results for the prevalence of severe depressive symptoms in older PLHIV. Among PLHIV aged 50 years or older living in Portugal, 23.9% presented chronic anxiety or depression [43]. In PLHIV aged between 56 to 65 living in the United States, the prevalence of severe depressive symptoms was 28.2% [27]. Data from SSA are scarce, presenting either the prevalence of a diagnosis of depression in PLHIV aged 50 years old or above or the age effects on depressive symptoms. In South Africa, the prevalence of major depressive disorders was 14.8% in PLHIV aged 50 years old or above on ART [44]. In rural South Africa, no significant difference was observed between age groups among PLHIV on ART but with a small sample size for the oldest (≥50 years) [45]. In other studies, younger age was associated with the presence of severe depressive symptoms but including a limited number of PLHIV aged above 50 years in Cameroun [46] or only middle-aged PLHIV in East Africa (mean age: 37.2 years) were included in those studies [14].

Compared to middle-aged PLHIV (median age < 45 years, 200 PLHIV included), and when using also the CES-D with the same cut-off as in our study, a prevalence of 18% was observed in Senegal [47]. In Nigeria and South Africa, using CES-D but with a cut off ≥16, the prevalence of severe depressive symptoms in middle-aged PLHIV (mean age < 45 years) on ART for at least six months was similar or higher (21 to 62%) [23, 48, 49]. In this context, we cannot conclude that older PLHIV are more likely to have severe depressive symptoms than younger ones. However, the lived reality of older PLHIV might be different from the one of the youngest.

Indeed, based on western countries’ studies, older PLHIV had to face different types of problems, including stigma [50] and specific concerns about disclosure due to age [51]. They also have some uncertainty about how aging, HIV, and long-term ART effects could interact and impact health [51]. The chronic aspect of the disease status and the increase of potential comorbidities with age could play an essential role in PLHIV related-depression, as observed in other chronic diseases [52]. Further studies are needed to better understand the reality of aging experience in PLHIV living in SSA.

Unemployment is commonly associated with depression or severe depressive symptoms in middle-aged PLHIV (mean age < 45 years) living in SSA [23, 31, 46, 53]. In South Africa, it was shown that unemployed PLHIV could have a 3-fold risk to present severe depressive symptoms and more than a 2-fold risk to be non-adherent to ART [23]. Even if we did not document any income information, being unemployed is often indirectly related to a lack of income, and so poverty. Acting as a stressor, low income could lead to difficulties in supporting health expenses, particularly the one due to other comorbidities, which are more likely to be numerous when PLHIV are getting older. Concerning retirement, we did not collect information about pension, limiting our conclusions.

Prior studies have documented an association between current cigarette smoking or nicotine dependence and the presence of severe depressive symptoms in middle-aged PLHIV living in western countries [54–56] but also with a diagnosis of major depression in older PLHIV living in Brazil [57]. As older PLHIV living in SSA are more likely to be regular smokers, and as tobacco consumption is often under-estimated [58], it is essential to screen depression in those patients. Even though few data are available about previous smokers among older PLHIV, those individuals might also be vulnerable and should be screened for depression.

The association between BMI and severe depressive symptoms in PLHIV is not systematically explored and makes no consensus [25, 59–61]. A low BMI could be an indirect marker of loss of appetite, one of the most reported symptoms in HIV related-depression. A low BMI could also be a marker of advanced disease, and in the social representation, HIV infection and mental illness are also often associated with thinness. Being overweight or obese could be in some ways protective against bad mood or stigmatization, but further investigations are needed to depict this point.

As observed in other studies conducted in SSA in middle-aged PLHIV, no effect of gender was observed [20, 24, 47]. However, the expression of depressive symptoms seems to be different in women and men, which is essential for clinicians to identify depressed patients.

In regard to HIV clinical data, a longer duration of the disease and a lower Nadir CD4 are associated in the univariate model with severe depressive symptoms. The impact of physical and emotional difficulties on the lived HIV experience should not be underestimated. In addition to the actual problem of living with HIV, long-time survivors might have to face different problems compared to those diagnosed more recently (i.e., confusion about surviving so far, a mourning of friends or family members lost to AIDS) [62]. The link with viral load could not be further investigated in our sample because of a significant proportion of missing data.

As depression has deleterious effects on PLHIV but is a modifiable condition, we encourage the screening and the management of depression in older PLHIV living in SSA. Promising results from a culturally-sensitive psychotherapeutic intervention [63] or a group-based counseling intervention [64] using task-shifting in middle-aged PLHIV living in SSA have already been reported. As older PLHIV are less likely to be engaged in behavioral health treatment for depression than younger PLHIV [65], it is essential to adapt psychotherapeutic interventions to the older PLHIV specific needs.

This study’s major strength is the opportunity to describe the prevalence of severe depressive symptoms in a large sample of older PLHIV, included in 3 different sites in West Africa. However, the generalizability of the results and the comparison with other studies could be limited as we included PLHIV on ART for at least six months within hospital-based study sites from urban settings. Second, the presence of depressive symptoms has been evaluated in the literature with diverse tools with different psychometric validities, leading to substantial variability in the measurements [7], hence results may not be generalizable across tools. Third, considerable stress related to HIV (i.e., HIV-related stigma, disclosure concerns, ART, physical changes) are essential determinants of depression and should not be underestimated even in older PLHIV. Further studies are needed to depict this. Fourth, even high pooled sensitivity and specificity was observed for the CES-D scale in African studies [35], a full clinical evaluation was not included in our scientific protocol to validate a major depressive disorder. As an interviewer-administered approach was used, and despite the staff’s full training, social-desirability bias might not be completely avoided. Finally, the cross-sectional design of the present study cannot provide information on the causal direction.

## Conclusions

The prevalence of severe depressive symptoms is high among older PLHIV living in West Africa, representing a severe problem for the organization of care and follow-up of PLHIV.

Unemployment and tobacco use were the main factors associated with severe depressive symptoms. Those patients should be considered as vulnerable and requiring additional support. Further studies in older PLHIV are needed to describe the phenomenon in more details and to better understand the reality of aging experience in PLHIV living in SSA. To guarantee the achievement of the 3 × 90 objectives, and encourage successful aging in PLHIV in West Africa, efforts are needed to integrate screening and management of depression in the standard of care. Finally, psychotherapeutic intervention adapted to older PLHIV specific needs should be developed.

## Data Availability

Complete data for this study cannot be posted in a supplemental file or a public repository at this current time because of scientific reasons here explained. This cross-sectional analysis is part of a 2-year longitudinal study evaluating different aspects of aging with HIV (cognition, physical function, and frailty) and the presence of severe depressive symptoms with a 2-year follow-up, which ended in December 2019. Cross-sectional analyses on cognition and frailty and longitudinal analyses on these topics, including depression, are currently in progress, and thus, data could not be posted at this time.

## Abbreviations

ADL: Activities of Daily Living
AIDS: A*cquired Immune Deficiency Syndrome*
aOR: Adjusted Odd Ratio
ART: Antiretroviral Therapy
BMI: Body Mass Index
CDC: Centers for Disease Control and Prevention
CES-D: Center for Epidemiological Studies Depression
CI: Confident Interval
HIV: Human Immunodeficiency Virus
IADL: Instrumental Activities of Daily Living
IeDEA: International epidemiological Databases to Evaluate AIDS
IQR: Interquartile Range
OR: Odds Ratio
PLHIV: People Living with HIV
SSA: Sub-Saharan Africa
